# Remission of necrobiotic xanthogranuloma after intravenous immunoglobulin interruption: A case report

**DOI:** 10.1177/2050313X241274888

**Published:** 2024-08-19

**Authors:** Amélie Gilbert-Falardeau, Anne-Marie Drolet

**Affiliations:** 1 Department of Dermatology, Université Laval, Québec, Québec, QC, Canada; 2 Department of Dermatology, Hôtel-Dieu de Lévis, Lévis, Québec, QC, Canada

**Keywords:** Necrobiotic xanthogranuloma, non-Langerhans cell histiocytosis, intravenous immunoglobulin, systemic therapy

## Abstract

Necrobiotic xanthogranuloma is a rare non-Langerhans cell histiocytosis with the potential for multisystemic involvement. It’s a challenging disease to treat and multiple treatments have been reported in the literature with variable results. We present the case of a 92-year-old woman with multiple orange-brown papules and plaques on her face progressing for several years. The biopsy showed dermal xanthogranulomatous infiltration with multinucleated giant cells and necrobiosis, and she was diagnosed with necrobiotic xanthogranuloma. A complete response was obtained with intravenous immunoglobulins and she remained in remission despite treatment discontinuation.

## Introduction

Necrobiotic xanthogranuloma is a rare disease that was first described by Kossard and Winkelmann in 1980.^1^ The disease is characterized by multiple yellow-orange plaques or nodules often in a periorbital distribution. Extracutaneous involvement of the eyes and other organs is possible and there is a close association with paraproteinemia and the development of multiple myeloma. Histopathology showed palisading granuloma with necrobiosis in the dermis/subcutis with Touton giant cells and cholesterol clefts.^2^ Treatments are based on case reports from the literature and intravenous immunoglobulins (IVIg) seem to be the most promising treatment. To our knowledge, this is the eighth case of complete response after IVIg and the first case to report remission after interruption of treatment.^3–9^

## Case

**Figure 1.**
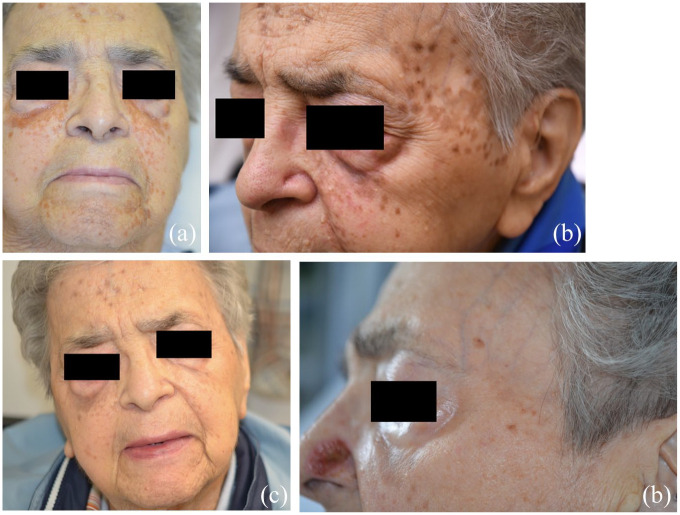
(a) At baseline. (b) After two cycles of IVIg, partial response with post-inflammatory brownish macules. (c) After seven cycles of IVIg, complete response. (d) Five months after discontinuation of IVIg, patient still in remission. IVIg: intravenous immunoglobulins.

A 92-year-old woman first presented to the dermatology department in May 2022 with multiple orange-brown papules and plaques on her forehead for more than 10 years. In the last 2 years, similar lesions have appeared on her periorbital skin, temples, cheeks, and chin (Figure 1(a)). No lesions were noted elsewhere on her body. The patient complained of mild pruritus but was mostly bothered by the aesthetic appearance of the lesions. The patient was known for hypertension, dyslipidemia, iron deficiency anemia, chronic kidney disease, and calcium pyrophosphate crystal deposition disease on colchicine and low-dose prednisone. She wasn’t known to have a hematological disease.

A biopsy of a forehead papule was obtained and showed a dermal xanthogranulomatous infiltration with multinucleated giant cells of Touton and foreign body types, focal necrobiosis, cholesterol clefts, lipid vacuoles, and lymphoid infiltrates. Laboratories revealed the presence of an IgG kappa paraproteinemia. A diagnostic of necrobiotic xanthogranuloma was made based on clinical, laboratory, and histopathological findings. Our case also fulfilled the proposed diagnostic criteria of a systematic review recently published.^2^

The patient was then referred to hematology and oncology for further investigations to rule out multiple myeloma. After discussion with the hematology team, the patient refused to undergo imaging and bone marrow biopsy because she didn’t wish to have treatment in the event that multiple myeloma was confirmed.

An initial treatment of intralesional triamcinolone (10 mg/ml) was attempted on a papule and showed no response. As the patient was very bothered by the appearance of the lesions on her face, IVIg 0.5 g/kg/day for four consecutive days every 4 weeks was administered. The dose administered was based on several case reports from the literature.^3,4,6–9^ After two cycles, most active lesions have disappeared with the presence of post-inflammatory brownish macules (Figure 1(b)). After seven cycles, there was complete resolution of the lesions and IVIg was discontinued (Figure 1(c)). The serum-free kappa light chains did not decrease with treatment. Five months after treatment discontinuation (12 months after starting IVIg), the patient was still in remission (Figure 1(d)). After the interruption of IVIg, the patient was diagnosed with a pseudogout flare versus polymyalgia rheumatica and was started on prednisone for 8 weeks and methotrexate. Unfortunately, the patient died of methotrexate toxicity making longer-term follow-up impossible.

## Discussion

Necrobiotic xanthogranuloma is a rare systemic disease of the seventh decade of life with a predilection in women (62.6%).^2^ The clinical features and associations described in our patient are consistent with the literature. The majority of patients have multiple, yellow or orange, plaques/papules on the periorbital area followed by other areas of the face, trunk, and extremities.^2^ Associated symptoms are common such as pain and pruritus.^2^ Ulceration, telangiectasia, atrophy, and induration are possible secondary features.^2^ Serum protein electrophoresis must be requested for all patients because paraproteinemia will be found in 82.1% (mostly IgG kappa).^2^ Some patients have associated neoplasia such as multiple myeloma, lymphoma, and leukemia.^2^ Multiple extracutaneous organs may be affected with the eye being the most commonly reported in 14.5% of patients.^2^

Treatment of necrobiotic xanthogranuloma is difficult and many systemic therapies have been reported in the literature with variable response. Due to the rarity of the disease, no controlled trials that evaluate systemic treatments have been done. A recent systematic review of systemic therapy by Steinhelfer et al. showed that the most effective treatment was IVIg followed by lenalidomide +/− corticosteroids and corticosteroids alone.^10^ Complete and partial response was achieved in 27% and 54% of patients treated with IVIg, respectively.^10^ The first case of successful treatment with IVIg was reported byHallermann et al. in 2010 and multiple cases treated with IVIg followed thereafter with variable response.^3^ To our knowledge, there are only seven cases in the literature with complete response to IVIg and our patient is the eighth described.^3–9^ Follow-up data were available for two patients with complete response and six patients with partial response and the median duration of response was 12 months as in our patient.^10^ Some authors reported sustained remission after extending intervals of IVIg to more than 4 weeks, but none have reported remission after interruption of IVIg.^3,4,6^ We are the first to describe a patient to be in remission 5 months after discontinuation of IVIg treatment. Although the short duration of prednisone received after IVIg didn’t contribute to the resolution of lesions, there is insufficient hindsight to know whether it helped maintain remission.

The pathogenesis of necrobiotic xanthogranuloma is unknown as is the effect of IVIg on the disappearance of lesions. We know that IVIg have anti-inflammatory and immunoregulatory effects and they are used to treat a variety of inflammatory and autoimmune diseases.^8^ Several hypotheses have been proposed to explain the mechanisms of action of IVIg such as B/T lymphocyte modulation, neutralization of pathological antibodies and inhibition of complement.^3,8^ IVIg is well-tolerated with the main adverse reaction being headache.^3^

In conclusion, necrobiotic xanthogranuloma is a challenging disease to treat and IVIg seems to be the treatment with the best response rate.^10^ Our case supports the efficacity of IVIg to achieve a complete response and to maintain remission even after discontinuation. More studies are needed to evaluate the best timing for treatment discontinuation and to follow long-term response.
